# Rhabdomyolysis and Acute Renal Failure Associated with Oxytetracycline Administration in Two Neonatal Foals Affected by Flexural Limb Deformity

**DOI:** 10.3390/vetsci7040160

**Published:** 2020-10-22

**Authors:** Nicola Ellero, Francesca Freccero, Aliai Lanci, Maria Morini, Carolina Castagnetti, Jole Mariella

**Affiliations:** 1 Department of Veterinary Medical Sciences (DIMEVET), University of Bologna, Via Tolara di Sopra 50, Ozzano dell’Emilia, 40064 Bologna, Italy; nicola.ellero3@unibo.it (N.E.); aliai.lanci2@unibo.it (A.L.); maria.morini@unibo.it (M.M.); carolina.castagnetti@unibo.it (C.C.); jole.mariella2@unibo.it (J.M.); 2 Health Science and Technologies Interdepartmental Center for Industrial Research (HST-ICIR), University of Bologna, 40064 Bologna, Italy

**Keywords:** neonatal foal, flexural deformity, oxytetracycline, rhabdomyolysis, acute renal failure

## Abstract

Oxytetracycline (OTC) administration has become a frequent practice in equine neonatology for the treatment of flexural limb deformity. The cause of this condition remains unclear but clinical studies revealed that following IV administration of OTC a relaxation of the metacarpophalangeal joint occurs in foals affected by flexural deformity. Studies concluded that OTC administration in neonatal foals did not adversely affect the kidneys. Other adverse effects of OTC have never been reported. This report presents two cases with different outcomes of 3-day-old foals which presented acute collapse and progressive depression after OTC administration. The clinical aspects, the increased activity of serum enzymes indicative of muscular damage, the presence of myoglobin in urine were clear diagnostic indicators of severe rhabdomyolysis, and the gross and histological findings confirmed a myopathy associated with renal damage in one case. Adverse effects on the musculoskeletal and urinary systems in healthy foals were first reported and were probably associated with multiple doses administered to foals less than 24–48 h old and/or at dosing intervals less than 24–48 h. The risk of development of rhabdomyolysis and nephrotoxicity in neonatal foals treated with OTC for flexural deformity from now on should be considered.

## 1. Introduction

Flexural deformity represents a deviation of a limb in the sagittal plane and is expressed as a persistent hyperflexion of a joint region [1]. Although the cause of this condition remains unclear, results of clinical and experimental studies revealed that following IV administration of oxytetracycline (OTC) a “relaxation” of the metacarpophalangeal joint angles occurs in foals with “contracted tendons” [2,3,4]. An in vitro study has suggested that OTC, through the calcium chelation, may inhibit the contraction of myofibroblasts within the flexor tendon itself [5]. In addition, OTC may be responsible for the inhibition of collagen remodeling during rapid growth of myofibroblasts through downregulation of the matrix metalloproteinase-1 gene expression; such that would increase check ligaments and tendons susceptibility to creep [6].

The reported dose of OTC for flexural deformities is 2.5–3 g regardless of bodyweight (bwt) (70 mg/kg SID IV in 30 min for 2–3 days, diluted in 500–1000 mL of 0.9% saline solution) [2].

Oxytetracycline administered IV at high doses is potentially nephrotoxic and may increase the risk of acute renal failure (ARF) in calves [7,8,9]. Studies evaluating the renal effects of OTC in healthy neonatal foals concluded that the medication did not adversely affect the kidneys [3,10]. Only one case of OTC-induced renal injury was reported in a 4-day-old foal that had concurrent neonatal isoerythrolysis [11]. To date, other adverse effects of OTC have not been reported.

This report describes clinical features and management of adverse reactions associated with OTC administration in two foals treated for flexural limb deformities.

## 2. Clinical Description

Data concerning the alterations observed in the presented cases for serum biochemistry, arterial blood gas analysis, electrolyte concentrations and urinalysis are reported in Table 1.

Case 1

In August 2017, a 3-day-old Italian Saddlebred colt, 45 kg bwt, was referred to the Perinatology and Reproduction Unit (Equine Clinical Service, Department of Veterinary Medical Sciences) of the University of Bologna for mild signs of meconium impaction and unilateral metacarpophalangeal flexural deformity. At admission, hematobiochemical parameters were within normal limits. The impaction was resolved with a single retention enema. Twenty-four hours from admission, the colt was treated with a single dose of 2 g (44.4 mg/kg) of oxytetracycline chlorhydrate IV slowly (TERRAMICINA 100, Zoetis Italia S.r.L., Rome, Italy), diluted in 500 mL of 0.9% saline solution, in order to treat the flexural deformity (off-label use). Four hours post-treatment, the foal showed acute collapse, depression, hyperthermia (39.3 °C), tachycardia (132 bpm) and tachypnea (96 breaths/min). Serum biochemistry showed an increase in creatine kinase (CK), aspartate transaminase (AST), lactate dehydrogenase (LDH) and urea. Arterial blood gas analysis revealed a mixed acidosis with hypoxemia, hypercapnia and decrease in bicarbonates. Lactatemia was at the upper normal limit. Electrolyte imbalances included hypokalemia and hypocalcemia. Urinalysis, including assessment of urine specific gravity, reagent strip analysis and sediment examination, showed brown colored and turbid urine, with USG 1016 and pH 7. Proteinuria, hemoglobinuria/myoglobinuria, glycosuria, and myoglobin casts were also present.

To provide respiratory support, intranasal oxygen therapy was started at 3–5 L per minute (LPM). Broad spectrum antimicrobial therapy was started with sodium ampicillin (50 mg/kg QID iv) and amikacin sulfate (30 mg/kg SID iv). Fluid therapy included Lactated Ringer’s solution at 5.6 mL/kg/h, supplemented with 20 mEq/L of potassium chloride, and 20% gluconate calcium solution at 2.0 mg/kg/h. To provide enteral nutrition, the foal was raised up and carried to the udder every hour. In 72 h, the clinical condition and the hematobiochemical parameters progressively improved, with the exception of elevated serum enzyme activity of CK, AST and LDH. Urinalysis was normal, with the exception of a mild hemoglobinuria/myoglobinuria. After 7 days of hospitalization, the colt was discharged.

Case 2

In February 2020, a 3-day-old Quarter Horse filly, 53 kg bwt, was referred to the same Unit due to acute onset of depression and anorexia. Before breeding, 5-panel genetic tests for the following disorders had been performed in both sire and dam (Laboklin veterinary clinical diagnostic laboratory, Deutschland, Steubenstraße 4, D-97688 Bad Kissingen): polysaccharide storage myopathy (PSSM), glycogen branching enzyme deficiency (GBED), hereditary equine regional dermal asthenia (HERDA), hyperkalemic periodic paralysis (HYPP) and equine malignant hyperthermia (EMH). At 8 h of life, in order to treat a bilateral metacarpophalangeal flexural deformity, the referring veterinarian had administered the filly 0.7 g (13.2 mg/kg) of oxytetracycline chlorhydrate IV (TERRAMICINA 100, Zoetis Italia S.r.L., Rome), diluted in 500 mL of 0.9% saline solution, SID for three consecutive days (off-label use).

At hospital admission, the foal was recumbent and depressed, with conserved sucking reflex. The rectal temperature was 35.6 °C. Heart (100 bpm) and respiratory (28 breaths/min) rates were within normal range but the foal presented weak peripheral pulse, cool limbs and pale oral mucous membranes, with prolonged capillary refill time (2.5 sec). Venous blood was collected from the jugular vein for blood culture, hematology, biochemistry and serum IgG determination, while arterial blood was collected from the metatarsal artery for blood gas analysis. Serum biochemistry showed a severe increase in CK, AST, LDH, urea, phosphorus and magnesium. Hypoglycemia was also present. Serum amyloid A concentration was 252 μg/dL. Blood gas analysis revealed a metabolic acidosis with hypocapnia, decrease in bicarbonates and hyperlactatemia. Electrolyte imbalances included hypocalcemia, chloride and sodium at the lower normal limit. Urinalysis showed brown colored and turbid urine, with USG 1012 and pH 5. Proteinuria and hemoglobinuria/myoglobinuria were also present. Transabdominal ultrasound of the urinary tract revealed abnormalities of the renal parenchyma, including a mild increased echogenicity, a mild loss of a distinct corticomedullary junction and a distinct curvilinear hyperechoic band in the outer renal medulla parallel to the corticomedullary junction. The bladder contained an inhomogeneous, swirling and echogenic fluid.

Broad spectrum antimicrobial therapy was started with ceftiofur sodium (10 mg/kg TID IV in 20 min). Fluid therapy included 10% dextrose at 3.4 mL/kg/h, supplemented with 20 mEq/L of potassium chloride, and 0.9% saline solution at 2.8 mL/kg/h. Twenty percent gluconate calcium, diluted in 0.9% saline solution, was also supplemented at 2.0 mg/kg/h. Enteral nutrition by nasogastric tube was started with 100 mL/h of mares’ milk (5% of foals’ bodyweight). In addition, the antioxidants vitamin E (1.4 IU/kg SID iv) and selenium (0.02 mg/kg SID IV slowly) were administered.

In 24 h, the clinical condition progressed to severe depression, hyperthermia (40.1 °C), tachycardia (196 bpm), dyspnea (60 breaths/min), muscle fasciculations, hypersalivation, sweating, abdominal distention, gastroesophageal reflux, oliguria and an early stage of pulmonary edema. Hypoxemia, hyperglycemia and hyperlactatemia were also present. Despite treatments, hematobiochemical parameters did not improve, especially hypocalcemia and serum enzyme activity of CK and LDH. Proteinuria and hemoglobinuria/myoglobinuria were still present and myoglobin casts were highlighted during urine sediment examination. To provide respiratory support, intranasal oxygen therapy was started at 3–5 LPM. Total parenteral nutrition (TPN) at 1–2 mL/kg/h and insulin infusion at 0.025–0.08 IU/kg/h were initiated. An association of dopamine (1–5 µg/kg/h) and furosemide (1 mg/kg/h) in continuous rate infusion was started to treat oliguria and pulmonary edema and midazolam infusion (1 mg/kg/h) was started to control muscle fasciculations. Despite additional treatments, the condition deteriorated to cyanotic mucous membranes, tetanic spasms, synchronous diaphragmatic flutter, convulsions, coma and death after 54 h of hospitalization.

**Figure 1:**
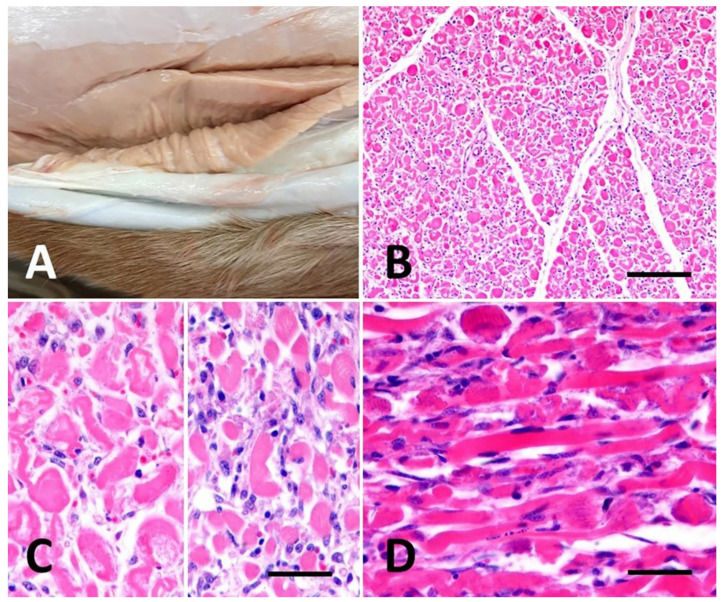
Gluteus muscle, case 2. (**A**) Grossly, severe pale pink discoloration on cut section. (**B**) Histological cross sections of skeletal muscle. Hematoxylin & eosin (H&E). At low power, marked fragmentation, swelling and degeneration of skeletal muscle fibers and infiltration by small dark cells (identified at higher magnification as proliferating satellite cells) were observed. Scale bar, 500 μm; (**C**) At higher magnification, a cross section showing lysis and degeneration of myofibers (left), proliferating satellite cells and infiltrations of macrophages (right). Scale bar, 200 μm; (**D**) longitudinal section showing severe muscle fiber degeneration and multifocal mineralization. Scale bar, 100 μm.

At necropsy, a diffuse pale pink to white discoloration of skeletal muscle in all muscle groups, including the diaphragm, was observed (Figure 1A). The heart also exhibited a diffuse tan discoloration. The subcutaneous tissues of the ventral abdomen and proximal hind limbs were diffusely thickened, wet and gelatinous and exuded copious amounts of clear fluid on cut section (edema). Lung tissue was rubbery and partially collapsed. The trachea and bronchi were filled with clear to white foam. The other organs appeared macroscopically normal. Histologically, muscle sections showed extensive areas of myofiber necrosis exhibiting sarcoplasmic fragmentation, hypercontraction of sarcomeres, hypereosinophilia, proliferation of satellite cells, nuclear pyknosis or karyolysis and infiltration with moderate numbers of macrophages. There was multifocal mineralization of necrotic myofibers and regeneration of myofibers characterized by centralization and rowing of satellite cell nuclei (Figure 1B–D). A Periodic Acid Schiff additional stain revealed no accumulation of subsarcolemmal glycogen within examined muscle sections. In the heart, 55% of sections microscopically examined showed cardiomyocyte necrosis characterized by loss of cross-striations, fragmentation, cellular vacuolization, nuclear pyknosis and sarcolemmal sheath collapse. Mild renal tubular degeneration and necrosis were present in the renal cortex, characterized by individual hypereosinophilic cells with loss of nuclear detail, sloughed pyknotic cells within the tubular lumen or the occasional presence of flattened epithelial cells lining renal tubules. Histologically, the lungs showed a severe and diffuse edema and congestion.

## 3. Discussion

Oxytetracycline is 50% protein bound and is well distributed to most tissues in horses, including the musculoskeletal system [17]. The volume of distribution is greater in neonatal foals (2 L/kg, [18]) than adult horses (0.34 to 0.95 L/kg, [19]). Oxytetracycline is eliminated in urine unchanged primarily by glomerular filtration, reaching relatively high concentrations, with peak above 1500 µg/mL [20]. Unmetabolized drug is also eliminated in the bile into the GI tract and may undergo enterohepatic recirculation, prolonging its effects [21]. The clearance of OTC is greater in foals (3.3 mL/min/kg, [18]) than adult horses (2.2 mL/min/kg, [22]) and if administered iv, the elimination half-life is longer in foals (7 h, [18]) than adult horses (6 h, [22]). Renal tubular necrosis caused by OTC is associated with high doses, outdated parenteral products, concurrent endotoxemia, dehydration, hypovolemia and pigment nephropathy [11]. Reportedly, in otherwise healthy foals, a single high-dose IV OTC administration for correction of flexural deformities does not cause renal toxicity [10], except for a case of oliguric renal failure developed in a foal with concurrent neonatal isoerythrolysis, which was given 70 mg/kg of IV OTC [11]. Adverse effects of OTC administration on muscles in foals have never been reported.

In the first reported case, a single IV OTC administration of the high (off-label) dose employed for flexural deformities resulted in acute collapse and subsequent weakness, recumbency and difficulty rising. This was probably attributed to intravascular chelation of calcium, systemic hypotension/hypoxemia, or both, which led to an initial stage of rhabdomyolysis. Pretreatment with IV calcium borogluconate could have avoided collapse [23,24]. In the second reported case, multiple high doses of the same drug resulted in recumbency, muscle fasciculations, trismus, hypersalivation, profuse sweating, tachycardia, tachy/dyspnea, hyperthermia, synchronous diaphragmatic flutter, convulsions, stupor, coma and death. This reaction was probably due to chelation of intracellular calcium and resulting neuromuscular blockade. In addition, the in vitro ability of OTC to inhibit tractional collagen structuring appears to be related to the dose-dependent inhibition of MMP-1 gene expression by equine myofibroblasts [6]. The inhibition of MMP-1 gene expression during this time may affect the remodeling process and make the developing tissue more susceptible to the neuromuscular blocking effect.

The clinical aspects, the increased activity of serum enzymes indicative of muscular damage, such as CK, AST and LDH, the presence of myoglobin in urine and the gross and histological findings (case 2) were clear diagnostic indicators of a severe rhabdomyolysis [25]. Urinalysis was the fastest means to detect myoglobinuria indicative of rhabdomyolysis, in both cases. Myoglobinuria has been detected by visual inspection of urine color and positive urine Hemastix test in the absence of hemolysis or hematuria. There are many differential diagnoses for skeletal muscle necrosis in horses, due to a variety of infectious agents, nutritional deficiencies, toxicities, immune-mediated and inherited metabolic disorders, that cause similar macroscopic findings.

Inherited metabolic disorders, such as polysaccharide storage myopathy (PSSM), cause myodegeneration and necrosis in horses [26]. The disease is characterized by an abnormal accumulation of glycogen and glycogen-related polysaccharide highlighted as Periodic Acid_Schiff-positive and amylase-resistant inclusions within skeletal muscle fibers [26]. Due to the absence of the accumulation of subsarcolemmal glycogen within the examined muscle sections (Periodic Acid Schiff-negative) and the genetic tests performed in both dam and sire in the second case, a presumptive diagnosis of PSSM was excluded.

Nutritional myopathy due to vitamin E and selenium deficiency, also known as white muscle disease (WMD), is typical of young foals and is expressed in two distinct cardiac and skeletal forms [27]. In the presented cases, the mares were fed with high-quality green forage during pregnancy, which brings an adequate vitamin E intake. In addition, no cases of muscle disorders were observed on the farm during the current breeding season or the previous ones. However, Vitamin E and selenium levels were not evaluated antemortem or postmortem in this study and therefore nutritional myopathy cannot be excluded definitively as a sole or contributing cause of the lesions.

Viral infections associated with myonecrosis in the horse include Equine Influenza, Equine Infectious Anemia and Equine Herpesvirus 1 [28]. In Italy, horses are routinely vaccinated for Equine Influenza and Equine Herpesvirus 1 and checked annually for Equine Infectious Anemia. A viral etiology was considered unlikely in this case, given the current vaccination status of their dams and the lack of supporting lesions in other organs in the second presented case. Several bacteria have been associated with equine rhabdomyolysis, especially clostridial organisms [29]. Albeit samples of muscle from the presented cases were not submitted for bacterial culture, the lack of compatible hematological and bacteriological (negative blood culture) findings, the gross and histopathologic appearance of the muscle lesions collectively ruled out bacterial infection as a potential diagnosis, including bacterial immune-mediated myopathy.

Numerous toxic agents are reported to cause myonecrosis in horses [30]. In light of the age of the foals and their permanence in separate straw-bedded boxes before hospitalization, toxic myopathy based on plants or feed additive ingestion was excluded from the list of differentials.

Myodegeneration associated with endotoxemia/systemic inflammatory response syndrome has been reported in the horse [31]. In the first presented case, the foal was primarily affected by a mild degree of meconium impaction, which certainly did not lead to an endotoxic status and a subsequent myonecrosis.

Based on the clinical history, the clinical progression and the gross and histologic findings in the presented cases, high-dose OTC administration-induced toxic myopathy represents the most probable diagnosis.

Both extrinsic, such as OTC-toxins, and intrinsic, such as excitation-contraction coupling, insults can cause rhabdomyolysis. However, they often share a final common pathway that leads to cell death-aberrant calcium cycling [32]. If the energy pathways that generate ATP for the calcium pump are impaired, if the calcium pump or channels are not functioning properly, or if the cell membrane is damaged, excessive calcium accumulates in the sarcoplasm. Mitochondria sequester excessive calcium but quickly become overloaded, disrupting oxidative metabolism, generating oxygen free radicals, activating proteases and phospholipases, and generating inflammatory cytokines, all of which combine to disrupt cellular architecture and function within the damaged segment of the myofiber [33].

Elevations in hundreds of thousands of IU/L and persistently elevated CK activity occurred in both cases, indicating a severe rhabdomyolysis and an ongoing muscle degeneration, respectively. Despite following damage to muscle cell membrane AST activity rises more slowly than CK, concurrent elevations in CK and AST reflect relatively recent or active degeneration [33].

Plasma concentration of extracellular electrolytes, such as sodium, chloride and calcium, were lower than normal in the presented cases. These ions move from blood into muscles following damage to muscle cell membranes, as occur with rhabdomyolysis. Conversely, plasma concentration of intracellular electrolytes, such as phosphorus and magnesium, were increased [34].

Severe rhabdomyolysis and OTC-toxins probably led also to renal compromise in the presented cases, due to the combined nephrotoxic effects of myoglobinuria and ischemia, respectively. Myoglobinuria, leading up to pigmentary nephropathy, has been also associated with the development of ARF in horses. Although the mechanism of pigment-induced renal injury is still not well understood, increased hydroxyl radical formation associated with the reduction of ferrous iron compounds, reduced renal blood flow caused by direct vasoconstrictor effects, and tubular obstruction by casts of heme proteins are certainly contributing factors. Myoglobin has also been suggested to induce renal vasoconstriction [35]. In the presented cases, only a mild increase in urea was highlighted at biochemistry, probably due to protein catabolism caused by fever and myositis, while no increase in creatinine was observed, despite urinalysis, clinical and histological findings were clear indicators of ARF [36]. However, it is well known how urea and creatinine are insensitive markers of renal function, specifically GFR, as they do not increase until about 75% of nephrons become nonfunctional [35]. Albeit monitoring creatinine over time increases its sensitivity in detecting changes in renal function [35], we did not detect an elevation. In the authors’ opinion, creatinine changes in these cases could be partly confounded by rapid elevations due to rhabdomyolysis and by fluid therapy, so should be considered with caution.

In the second case, the persistence of oliguria in the face of fluid administration and TPN led to fluid retention and initial development of pulmonary edema. Simultaneous infusion of dopamine and furosemide was then elected. While low-dose rate of dopamine may improve renal blood flow and urine output, the use of diuretic agents such as loop diuretics to treat ARF is controversial [37]. In the authors’ opinion, furosemide was indicated in the presented case, due to the capacity of promoting diuresis, reducing tubule cells’ metabolic rate and thus oxygen demand [38]. In addition, loop diuretics appear to be most beneficial when used in cases characterized by tubule obstruction, because the increase in solute retention apparently helps flush these blockages and casts from the tubules [39].

## 4. Conclusions

In conclusion, high-dose (off-label) OTC therapy has become a frequent practice in equine neonatology for the treatment of flexural deformity. In the present study, adverse effects on the musculoskeletal and urinary systems of healthy foals were first reported and were probably associated with multiple doses administered to foals less than 24–48 h old and/or at dosing intervals less than 24–48 h. In case 1, a single OTC dose was administered at 72 h of life, while, in case 2, multiple OTC doses were administered from 8 h of life. Despite appropriate initial administration in the first case and appropriate intervals between multiple administration in the second one, rhabdomyolysis and ARF were observed in both foals. Another intrinsic factor that could be considered is a breed-related predisposition in Quarter Horse foals.

The risk of development of rhabdomyolysis and nephrotoxicity in neonatal foals treated with IV OTC for flexural deformity from now on should be considered. In light of the novel information provided by the presented cases and the “One Health” concept introduced in the early 2000s, veterinarians should make more judicious use of antibiotics [40], especially off-label, and consider alternative therapies for the treatment of flexural limb deformities in neonatal foals. Mild congenital cases of metacarpophalangeal flexural deformity often self-correct. Usually, if the neonate is able to stand on the affected leg, no therapy is required and the foal will spontaneously improve over 4 to 5 days [41]. If the deformity is mild to moderate, in cases in which the limb is manually reducible, controlled exercise in combination with pain relief using nonsteroidal anti-inflammatory drugs (NSAIDs) may be sufficient, although more severe cases may require therapeutic farriery and/or immobilization (bandages and splints) [41]. When foals are not loading the heels, they may benefit from temporary heel support, which is gradually reduced. For deformities not responding to conservative treatment, a distal check ligament desmotomy should be considered in combination with NSAIDs and toe extension. In very severe cases, tenotomy of the deep digital flexor tendon may be required, although this generally results in the loss of athletic function [1].

## Figures and Tables

**Table 1:** Summary of alterations observed in the presented cases for serum biochemistry, arterial blood gas analysis, electrolyte concentrations and urinalysis.

		Case 1			Case 2		
		24 Hours Post-Admission; 4 Hours After Single OTC Dose	4 Days Post-Admission	7 Days Post-Admission	Admission; 12 Hours After 3rd OTC Dose	24 Hours Post-Admission	48 Hours Post-Admission
parameter	ref. range	serum biochemistry					
CK (IU/L)	52–143 [12]	62,300	4199	1238	1,000,000	999,999	102,000
AST (IU/L)	237–620 [12]	13,295	9894	3453	16,738	1	1
LDH (IU/L)	225–700 [12]	7136	1280	569	81,689	120,949	28,587
creatinine (mg/dL)	1.0–1.7 [12]	0.96	0.63	NA	1.18	1.14	NA
urea (mg/dL)	4–20 [12]	24.14	12.72	NA	32	59	NA
phosphorus (mg/dL)	5.4–9.4 [12]	NA	NA	NA	11.30	8.20	NA
magnesium (mg/dL)	1.12–1.85 [13]	2.23	2.10	NA	2.90	2.91	NA
SAA (μg/dL)	NA	NA	NA	NA	252	230	NA
		arterial blood gas analysis					
pH	7.36–7.39 [14]	7.28	7.36	NA	7.27	7.19	7.31
paO_2_ (mmHg)	84.7–89.1 [14]	61.6	84.5	NA	117	37.7	35.1
paCO_2_ (mmHg)	45.6–47.8 [14]	49.2	45.6	NA	35.7	41.7	48.9
HCO_3_ (mmol/L)	24.8–26.4 [14]	22.4	24.8	NA	16.5	14.6	21.9
lactate (mmol/L)	0.9–3.6 [15]	3.6	1.8	NA	15.1	15.3	17.4
glucose (mmol/L)	6.7–12.9 [14]	10.3	10.6	NA	1.1	14.1	10.3
		electrolyte concentrations					
sodium (mmol/L)	130–154 [12]	133	139	NA	130	140	142
potassium (mmol/L)	3.8–5.8 [12]	2.6	3.9	NA	4.3	3.2	3.1
chloride (mmol/L)	94–110 [12]	100	108	NA	94	89	97
total calcium (mmol/L)	2.8–3.4 [12]	1.14	1.18	2.8	0.86	0.58	0.76
		Urinalysis					
USG	1004–1008 [16]	1016	1, 12	NA	1012	1011	1012
pH	5.5–8.0 [16]	7	6.5	NA	5	5	5
proteins (mg/dL)	neg. [16]	500	neg.	NA	100	100	30
hemo/myoglobin (erythrocytes/μL)	neg. [16]	250	10	NA	250	250	250
glucose (mg/dL)	neg. [16]	100	neg.	NA	neg.	neg.	neg.
myoglobin casts	neg. [16]	pos.	neg.	NA	neg.	pos.	pos.

SAA: serum amyloid A; USG: urine specific gravity; NA: data not available. [12] Bauer, J.E.; Harvey, J.W.; Asquith, R.L.; McNulty, P.K.; Kivipelto, J.A.N. Clinical chemistry reference values of foals during the first year of life. Equine Vet. J. 1984, 16, 361–363. [13] Mariella, J.; Isani, G.; Andreani, G.; Freccero, F.; Carpenè, E.; Castagnetti, C. Total plasma magnesium in healthy and critically ill foals. Theriogenology. 2016, 85, 180–185. [14] Aguilera-Tejero, E.; Estepa, J.C.; Lopez, I.; Mayer-Valor, R.; Rodriguez, M. Arterial blood gases and acid-base balance in healthy young and aged horses. Equine Vet. J. 1998, 30, 352–354. [15] Castagnetti, C.; Pirrone, A.; Mariella, J.; Mari, G. Venous blood lactate evaluation in equine neonatal intensive care. Theriogenology. 2010, 73, 343–357. [16] Corley, K.T.T.; Stephen, J. Appendix. In The Equine Hospital Manual, 1st ed.; Corley, K.T.T., Stephen, J., Eds.; Blackwell: Oxford, UK, 2008; 654–689.
